# Pedicled Latissimus Dorsi Flap Vascularized by a Lumbar Artery Perforator for the Reconstruction of an Exposed Lumbar Spinal Fixation Device: A Case Report

**DOI:** 10.7759/cureus.78163

**Published:** 2025-01-28

**Authors:** Riko Sakaguchi, Kaoru Sasaki, Junya Oshima, Yukiko Aihara, Mitsuru Sekido

**Affiliations:** 1 Department of Plastic, Reconstructive, and Hand Surgery, University of Tsukuba, Tsukuba, JPN

**Keywords:** angiogenesis, indocyanine green fluorescence angiography, latissimus dorsi flap, lumbar artery perforator, pedicled flap, reconstructive microsurgery

## Abstract

Latissimus dorsi flaps are typically vascularized by the thoracodorsal or intercostal artery perforators, while cases describing the use of lumbar artery perforators are exceedingly rare. This report presents a case of a 74-year-old woman presenting with a refractory ulcer associated with an exposed lumbar spinal fixation device. Reconstruction was successfully performed using a pedicled latissimus dorsi flap vascularized by lumbar artery perforators. Preoperative indocyanine green (ICG) fluorescence angiography confirmed adequate perfusion from the lumbar artery perforators. Postoperative outcomes were favorable, with no ulcer recurrence observed after six months of follow-up. Chronic inflammation and malignancy were considered as likely contributors to angiogenesis and increased blood flow around the lumbar artery perforators. Additionally, ICG fluorescence angiography proved an effective and minimally invasive technique for evaluating flap perfusion.

## Introduction

The latissimus dorsi flap is a widely used option in reconstructive surgery, traditionally relying on the thoracodorsal artery pedicle or intercostal artery perforators as vascular pedicles [1]. The use of lumbar artery perforators to vascularize the latissimus dorsi flap is less common due to concerns regarding distal blood flow insufficiency. To date, only one reported case has documented the application of such a flap for reconstruction following resection of a squamous cell carcinoma originating from an untreated meningocele; however, there is no detailed description of the flap [2].

In the present case, the use of a free latissimus dorsi flap was initially planned for the reconstruction of a refractory ulcer with exposure of a lumbar spinal fixation device. However, poor vascular conditions at the planned recipient site were identified during intraoperative evaluation, necessitating a shift to a pedicled latissimus dorsi flap vascularized by lumbar artery perforators. Blood flow dynamics of the lumbar artery perforators and the flap were assessed in real-time using intraoperative indocyanine green (ICG) fluorescence angiography, allowing for appropriate surgical modification. This case highlights the potential for expanding reconstructive options using atypical pedicles, thereby laying the groundwork for future clinical applications.

## Case presentation

**Figure 1:**
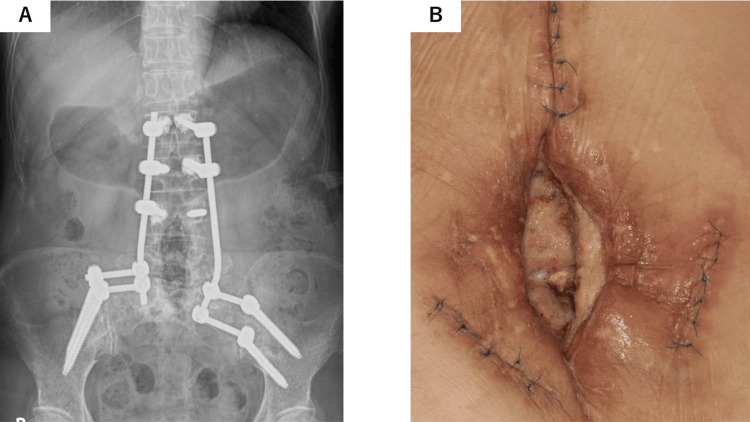
Clinical presentation of the ulceration and exposed spinal fixation hardware. (A) Spinal fixation with pedicle screws in the L1–3 region and iliac screws shown on radiographs. (B) Preoperative view of the refractory ulcer with exposed lumbar fixation hardware in the L4–5 region, showing a 6 cm × 12 cm defect.

A 74-year-old woman presented with a refractory ulcer following multiple spinal surgeries. Three years earlier, she had been diagnosed with a fifth lumbar vertebra (L5) chordoma and underwent maximal tumor resection with spinal pelvic fixation. Subsequent interventions included two interbody fusion procedures between the second and fourth lumbar vertebra (L2 and L4) and posterior fixation between the first and second sacral vertebra (S1 and S2) using the S2-alar-iliac technique. Proton beam therapy (35.2 GyE in 32 fractions) was administered to the region between the fourth and fifth lumbar vertebrae (L4 and L5); however, radiation dermatitis occurred as a side effect. Two months prior to presentation, spinal fixation was performed using pedicle screws in the region between the first and third lumbar vertebrae (L1 and L3), along with iliac screws (Figure 1A). This procedure resulted in a 6 cm × 12 cm defect with exposure of the lumbar fixation hardware and infection in the L4-L5 region (Figure 1B).

The refractory ulcer likely arose from a combination of radiation dermatitis and chronic hardware exposure, which complicated the wound healing process. Despite debridement and negative pressure wound therapy, wound healing was not achieved, and the patient was referred to our department for further management.

Surgical findings

**Figure 2:**
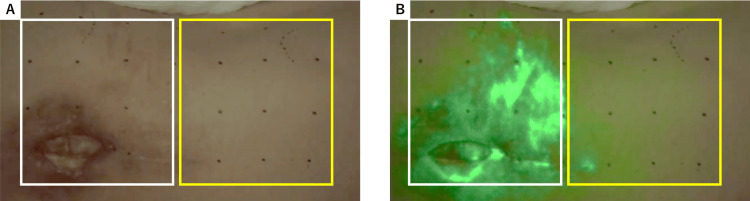
ICG fluorescence angiography for blood flow assessment. (A) Preoperative view prior to ICG staining. (B) ICG fluorescence angiography 30 seconds after staining shows faint and delayed staining in the intercostal artery perforator region (yellow square) but early and intense staining in the lumbar artery perforator region (white square), indicating robust blood flow dynamics.

The initial plan involved a free latissimus dorsi flap anastomosed to the superior gluteal vessels. Preoperative indocyanine green (ICG) fluorescence angiography revealed faint and delayed staining in the intercostal artery perforator region of the latissimus dorsi. In contrast, the lumbar artery perforator region and surrounding wound area demonstrated early and intense staining within 30 seconds, indicating adequate blood flow in the lumbar artery perforator region (Figures 2A, 2B).

**Figure 3:**
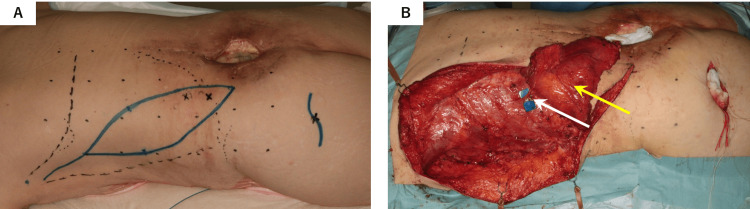
Surgical design and elevation of the pedicled latissimus dorsi flap and vascular pedicle preservation. (A) A 26 cm × 6 cm pedicled latissimus dorsi flap is marked, incorporating the lumbar artery perforators as the vascular pedicle. The incision line for superior gluteal artery access is also shown. (B) The latissimus dorsi flap (yellow arrow) could not reach the lumbar skin defect unless the ninth intercostal artery perforator (white arrow) was ligated and divided. The lumbar artery perforators and their surrounding vascular territories are preserved.

The 26 cm × 6 cm flap was marked to include these perforators (Figure 3A). The position of the left superior gluteal artery, the intended recipient artery, was identified preoperatively using ultrasound, and an incision line was designed directly above its location. During surgery, the superior gluteal vessels were found to be unsuitable due to insufficient caliber and flow. Consequently, the surgical plan was modified to use a latissimus dorsi flap vascularized by lumbar artery perforators, with the option for additional arterial anastomosis if necessary. The latissimus dorsi muscle was elevated in its entirety while preserving the origin of the lumbar artery perforators. The thoracodorsal vessels, as well as the ninth and tenth intercostal artery perforators, were ligated to facilitate flap mobilization (Figure 3B). 

**Figure 4:**
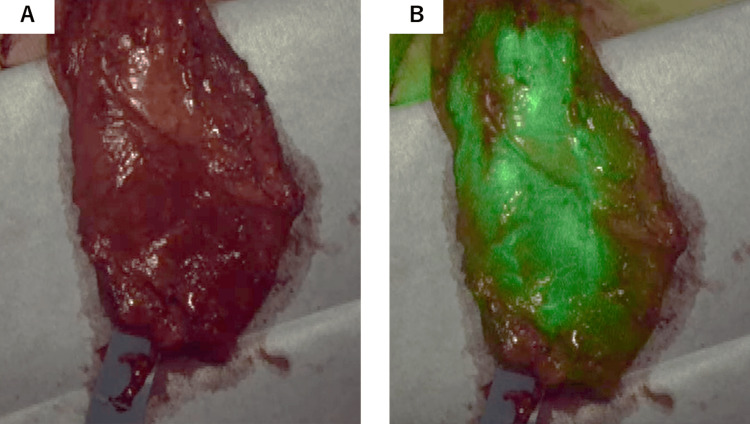
Intraoperative flap perfusion using ICG fluorescence angiography. (A) Post-flap-elevation view prior to ICG staining. (B) ICG fluorescence angiography 168 seconds after staining confirms perfusion to the distal latissimus dorsi flap based on lumbar artery perforators. However, the delayed peak fluorescence indicates suboptimal blood flow. The figure captures the moment when fluorescence reaches peak intensity.

The flap was then pivoted around the lumbar artery perforators, and its muscle bulk was folded back to fill the defect. Intraoperative ICG fluorescence angiography confirmed perfusion to the distal portion of the flap, which was supplied solely by the lumbar artery perforators and their surrounding tissues. However, fluorescence took 168 seconds to reach peak intensity, indicating that while blood flow was maintained, it was suboptimal (Figures 4A, 4B).

To mitigate potential tension or compression following flap placement, an additional arterial anastomosis was performed between the thoracodorsal artery and the contralateral right lumbar artery.

**Figure 5:**
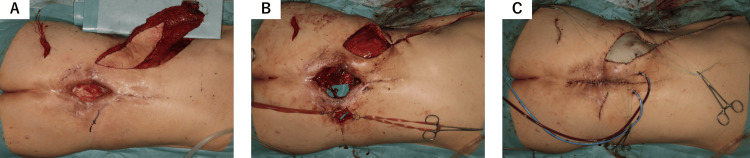
De-epithelialization, flap inset, defect coverage and final closure with skin grafting (A) The latissimus dorsi flap vascularized by lumbar artery perforators is elevated. (B) The de-epithelialized latissimus dorsi flap is folded back and inset into the lumbar defect, filling the wound with its muscle bulk. Subcutaneous tunneling is performed to ensure proper alignment and tension-free placement. (C) Split-thickness skin grafts are placed on the de-epithelialized skin of the latissimus dorsi flap to achieve complete defect closure. Primary sutures are used for final wound closure.

Debridement and irrigation of the defect were performed, with necrotic tissue thoroughly removed. A subcutaneous tunnel was created to position the latissimus dorsi flap, which was folded back and inset into the defect. The skin of the latissimus dorsi flap was de-epithelialized (Figures 5A, 5B). The left superior gluteal artery, initially deemed unsuitable as a recipient vessel, was repurposed as an interposed graft. Vascular anastomoses were completed using 10-0 nylon for the thoracodorsal artery (2 mm diameter) to the left superior gluteal artery (1 mm diameter) and 11-0 nylon for the left superior gluteal artery (1 mm diameter) to the right lumbar artery perforator (0.5 mm diameter). The skin surrounding the defect was approximated and closed with primary sutures. Exposed areas of the folded-back muscle were covered with split-thickness skin grafts derived from the de-epithelialized skin of the flap (Figure 5C).

**Figure 6:**
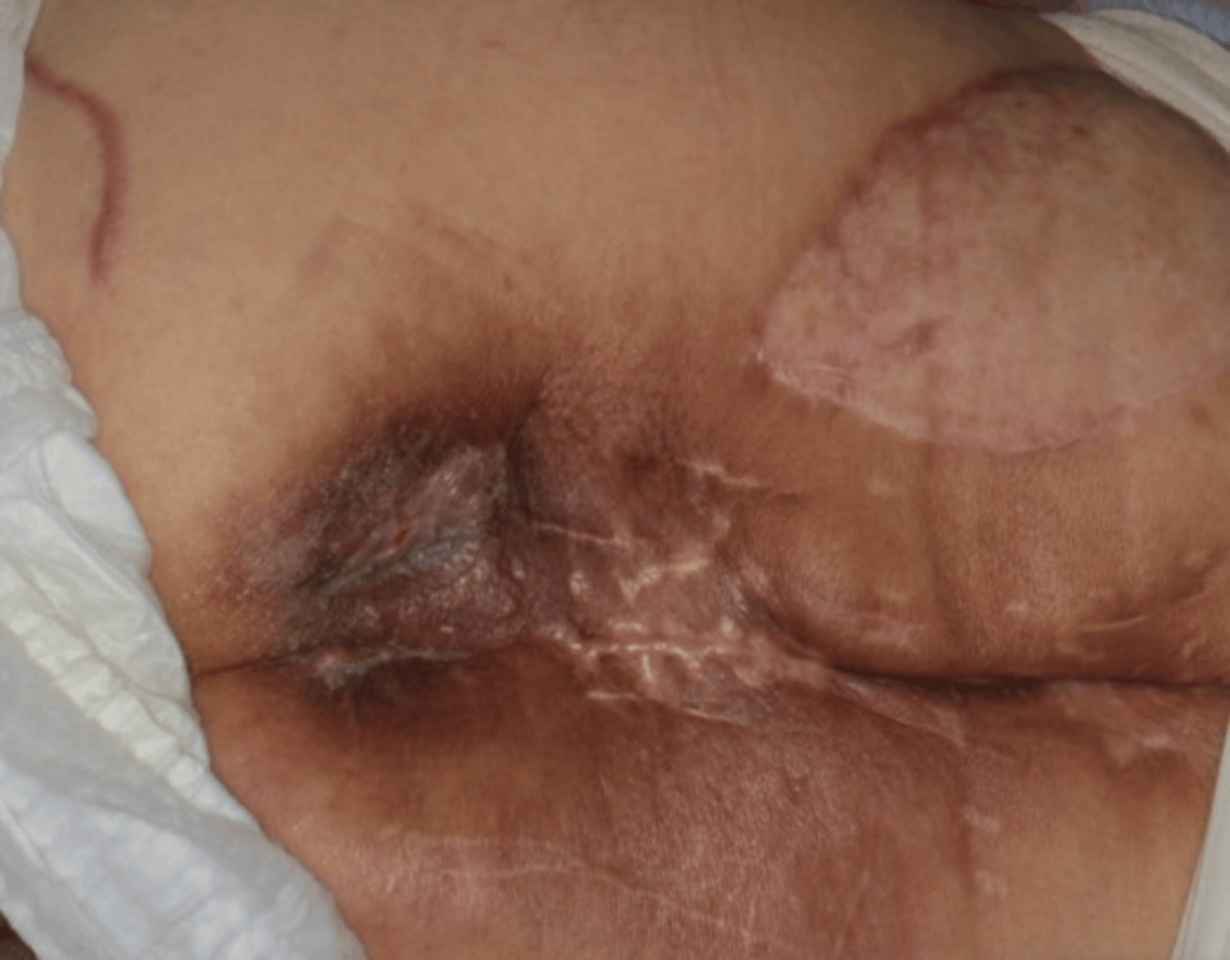
Six-month postoperative outcome. On follow-up, the reconstructed site shows a viable flap with no recurrence of ulceration or hardware exposure six months after surgery.

Postoperatively, the flap remained viable, and no recurrence of ulceration was observed during six months of follow-up (Figure 6).

ImageJ software (Rasband, W.S., ImageJ, U.S. National Institutes of Health, Bethesda, Maryland, USA) was used to measure ICG fluorescence angiography brightness. The videos were imported into ImageJ and converted into a time-stack format. Regions of interest (ROIs) were selected using the rectangular selection tool. In the preoperative ICG fluorescence angiography, three lumbar artery perforators were identified and defined as ROIs. In the ICG fluorescence angiography of the flap based solely on lumbar artery perforators, the ROI was set at the flap’s distal region. The brightness values within the ROIs were measured across the time stack using ImageJ’s standard functionality, and the data were exported as a CSV file for subsequent analysis.

## Discussion

The latissimus dorsi muscle is supplied by three vascular territories. Starting from the distal end, the first territory is supplied by the thoracodorsal artery and the ninth and tenth intercostal artery perforators. The second territory receives blood from the eleventh intercostal artery and the subcostal artery perforators, while the third territory is supplied by the first and second lumbar artery perforators. These territories are interconnected by a network of communicating vessels [3]. In contrast, the skin and subcutaneous fat of the back are supplied by two vascular territories. The first territory is nourished by the thoracodorsal artery, the ninth, tenth, and eleventh intercostal artery perforators, and the circumflex scapular artery perforators. The second territory is supplied by the subcostal artery and the first and second lumbar artery perforators [3].

In the case presented, early and intense staining of the area surrounding the lumbar artery perforators was observed during preoperative ICG fluorescence angiography, indicating increased blood flow. This observation is likely attributable to the effects of malignancy and chronic inflammation. Malignant tumors are well-documented to stimulate angiogenesis in adjacent tissues through mechanisms involving vascular endothelial growth factor (VEGF) and matrix metalloproteinase-9 (MMP-9). Specifically, chordomas have been reported to express elevated levels of VEGF and MMP-9 [4,5]. MMP-9 plays a role in releasing VEGF bound to the extracellular matrix [6], facilitating their interaction and promoting angiogenesis in the tumor-adjacent tissues, including the lumbar artery perforator region.

Furthermore, the multiple surgical interventions likely induced chronic inflammation in the wound area, driving the production of the inflammatory cytokines essential for angiogenesis. Key cytokines involved in this process include tumor necrosis factor-alpha (TNF-α) and interleukin-6 (IL-6) [6-8]. TNF-α activates macrophages and other immune cells, creating an inflammatory environment through the production of additional cytokines (e.g., IL-1, IL-6, IL-8) and promoting the expression of VEGF and MMPs in vascular endothelial cells [6,7]. IL-6 increases vascular permeability, enhancing nutrient and oxygen exchange between tissues while stimulating endothelial cells via the Janus Kinase/Signal Transducer and Activator of Transcription 3 (JAK/STAT3) signaling pathway to produce VEGF [6,7]. These combined mechanisms likely contributed to the increased blood flow around the lumbar artery perforators in this case. The pedicle of the latissimus dorsi flap not only included the lumbar artery perforators but also incorporated the adjacent origin of the latissimus dorsi, subcutaneous tissue, and dermal layers, where angiogenesis occurred and contributed to flap perfusion.

As demonstrated in this case, ICG fluorescence angiography is a valuable tool for evaluating flap perfusion and has broader intraoperative applications, such as lymph node dissection, intestinal anastomosis assessment, tumor boundary visualization in liver and kidney surgeries, and parathyroid gland identification in thyroid surgeries [9]. Its simplicity and minimally invasive nature are key advantages of ICG fluorescence angiography across these applications. In reconstructive microsurgery, ICG fluorescence angiography has been shown to be effective for perforator identification [10] and serves as a highly accurate predictor of flap necrosis [11,12].

In this case, real-time ICG fluorescence angiography enabled precise evaluation of blood flow dynamics in the lumbar artery perforators and the pedicled latissimus dorsi flap, enabling an appropriate surgical modification. Its application in atypical flaps may expand reconstructive options by providing a detailed assessment of perfusion. Currently, ICG fluorescence angiography primarily relies on qualitative and subjective assessments by surgeons, as real-time quantification remains technically challenging. Factors such as variations in camera angle and distance, as well as ICG dosage, contribute to the difficulty of standardizing quantification [13]. Advancements in real-time quantification techniques and standardized evaluation criteria hold significant promise for improving treatment precision and minimizing postoperative complications.

## Conclusions

Here, we reported a case involving a latissimus dorsi flap vascularized solely by lumbar artery perforators. Malignancy and chronic inflammation were likely important factors contributing to the enhanced blood flow observed around the lumbar artery perforators. ICG fluorescence angiography, a simple and minimally invasive technique, proved invaluable in guiding the selection of the appropriate treatment approach. This case demonstrates the feasibility of lumbar artery perforators as a viable alternative vascular pedicle in complex reconstructive surgeries, potentially expanding options for managing challenging cases.
